# Recomendaciones para la medición de esteroides sexuales en la práctica clínica. Documento de posicionamiento SEQC^ML^/SEEN/SEEP

**DOI:** 10.1515/almed-2022-0121

**Published:** 2023-02-24

**Authors:** Gregori Casals, Roser Ferrer Costa, Eulàlia Urgell Rull, Héctor F. Escobar-Morreale, Jesús Argente, Gemma Sesmilo, Betina Biagetti

**Affiliations:** Servicio de Bioquímica y Genética Molecular, Hospital Clínic, IDIBAPS, CIBEREHD Universidad de Barcelona, Barcelona, España; Servicio de Bioquímica, Laboratoris Clínics, Hospital Universitari Vall d’Hebron, Universitat Autònoma de Barcelona, Barcelona, España; Servicio de Bioquímica, Hospital de la Santa Creu i Sant Pau, Barcelona, España; Servicio de Endocrinología y Nutrición, Hospital Ramón y Cajal, Universidad de Alcalá, Instituto Ramón y Cajal de Investigación Sanitaria IRYCIS y CIBER Diabetes y Enfermedades Metabólicas Asociadas CIBERDEM, Madrid, España; Departamento de Pediatría y Endocrinología Pediátrica, Hospital Infantil Universitario Niño Jesús, Universidad Autonoma de Madrid, CIBEROBN, Instituto de Salud Carlos III, Madrid, España; Servicio de Endocrinología y Nutrición, Hospital Universitari Dexeus, Barcelona, España; Servicio de Endocrinología y Nutrición, Hospital Universitari Vall d’Hebron, Universitat Autònoma de Barcelona, Barcelona, España

**Keywords:** esteroides sexuales, testosterona, estradiol, inmunoensayo, espectrometría de masas

## Abstract

La correcta aproximación clínica a un amplio grupo de situaciones depende en gran medida de la disponibilidad de resultados analíticos de esteroides sexuales que sean exactos y reproducibles, obtenidos con métodos con la especificidad y sensibilidad analíticas adecuadas. En este sentido, los inmunoanálisis quimioluminiscentes actuales presentan limitaciones analíticas con repercusiones clínicas importantes. El documento de posicionamiento revisa el estado actual en la estandarización de los métodos de medida de estradiol y testosterona y su repercusión en distintas situaciones clínicas. Se incluye asimismo una serie de recomendaciones a seguir para introducir en los sistemas nacionales de salud los análisis de esteroides por espectrometría de masas, metodología recomendada desde hace más de una década por las sociedades internacionales.

## Introducción

Los esteroides sexuales son responsables del desarrollo y la maduración del individuo, además de intervenir en muchas otras funciones [1, 2]. Su desequilibrio se relaciona con alteraciones en el metabolismo, el hueso, y el desarrollo o progresión de ciertos tumores, entre otros trastornos.

A pesar de la gran importancia de los esteroides sexuales, nuestra capacidad para medir sus concentraciones correctamente varía ampliamente según el método.

La especificidad, sensibilidad, exactitud, precisión y la estandarización de la medición de estas hormonas es muy relevante para el manejo de un amplio grupo de situaciones clínicas. La obtención de resultados fiables nos influye en gran medida en la toma de decisiones, evitando diagnósticos y tratamientos erróneos y seguimientos innecesarios.

En este documento, discutimos temas relacionados con las mediciones de esteroides sexuales en cuanto a las diferentes técnicas de medición y su impacto en la práctica clínica, y proporcionamos unas recomendaciones consensuadas por las Sociedades Españolas de Medicina de Laboratorio (SEQC^ML^), Endocrinología y Nutrición (SEEN) y Endocrinología Pediátrica (SEEP).

## Evolución histórica de los métodos de medida

Las primeras técnicas de medida para el análisis de hormonas esteroides incluían distintos métodos químicos y bioensayos. Estos métodos presentaban una sensibilidad analítica limitada (concentraciones de miligramo o microgramo por litro) por lo que estaban restringidos mayoritariamente al análisis de esteroides conjugados en orina. El desarrollo del radioinmunoanálisis (RIA) posibilitó las primeras mediciones hormonales con suficiente sensibilidad (nanogramo o picogramo por litro) y especificidad en suero y plasma. El primer RIA data del 1959 [3], fue originalmente diseñado para hormonas peptídicas (insulina) y extendido diez años más tarde a moléculas más pequeñas como los esteroides, siendo el estradiol el primero en desarrollarse por Abraham et al. [4] El desarrollo e implantación de distintos métodos basados en RIA para el análisis de hormonas ha tenido un gran impacto en el desarrollo de la endocrinología moderna.

Los investigadores que desarrollaron los primeros inmunoanálisis para moléculas pequeñas eran conscientes de las dificultades y limitaciones de estos ensayos. De hecho, la tardanza en la aparición del primer inmunoanálisis para un esteroide vino motivada por la necesidad de establecer y optimizar pasos preanalíticos adicionales para crear inmunoanálisis que fueran válidos para moléculas pequeñas y poco inmunogénicas como los esteroides. Estos no son otra cosa que moléculas lipídicas originadas a partir del colesterol, generadas por un número limitado de transformaciones enzimáticas y, por lo tanto, son muy similares estructuralmente. Estos pasos preanalíticos comprendían esencialmente la extracción con solventes orgánicos y la cromatografía, permitiendo separar eficazmente los diferentes esteroides presentes en fluidos biológicos como el suero, el plasma o la orina. De esta forma se consiguió, ya en los años 1970, el análisis de esteroides en suero con buena sensibilidad y especificidad analíticas, aunque restringido a laboratorios especializados dado el importante procesamiento manual de las muestras. Las ventajas de los RIA con purificación previa de la muestra incluyen la eliminación de metabolitos potencialmente interferentes y la desnaturalización de las proteínas de transporte como la globulina transportadora de hormonas sexuales (SHBG) durante el proceso de extracción con solventes orgánicos, que libera los esteroides como testosterona o estradiol. También, tienen la posibilidad de adecuar el volumen de muestra a la sensibilidad esperada, y permiten medir diversos esteroides en una misma muestra tras separarlos según su peso molecular y polaridad mediante cromatografía.

Estos métodos de RIA con pasos previos de purificación de la muestra han contribuido de forma importante al conocimiento endocrinológico. En el campo del diagnóstico clínico han sido importantes en el correcto diagnóstico y tratamiento, y continúan siendo métodos válidos actualmente dado su buen comportamiento analítico cuando están bien validados. Sin embargo, presentan algunas desventajas derivadas de la necesidad de instalaciones para radioactividad, elevada laboriosidad y bajo rendimiento práctico (normalmente se tarda 2 días en medir un único esteroide en unas 40 muestras).

A finales de 1970, gracias al desarrollo de anticuerpos más específicos, y de técnicas de doble anticuerpo, se desarrollaron métodos de RIA directo para el análisis de hormonas esteroides que, a diferencia de los primeros RIAs, permitían omitir los pasos de purificación de la muestra previos al inmunoanálisis. En las décadas 1980–90, los marcadores radioactivos de los RIA fueron reemplazados por marcadores quimioluminiscentes, fluorescentes o enzimáticos que, coincidiendo con el gran aumento de la demanda analítica, facilitó la incorporación de kits de reactivo simplificados y automatizables, y permitieron una gran capacidad de análisis en término de cantidad muestras.

Actualmente los inmunoanálisis quimioluminiscentes directos (sin extracción previa de la muestra) son los más ampliamente utilizados para el análisis de las principales hormonas sexuales. Aunque sus limitaciones analíticas fueron identificadas rápidamente [5], no es hasta principios de los años 2000 donde éstas adquieren mayor relevancia debido, por un lado, al mayor grado de escrutinio de los métodos existentes y, por otro lado, a la creciente accesibilidad de métodos estructurales basados en espectrometría de masas. En este sentido, tuvo un papel destacado la publicación de Taieb et al. [6], que puso de manifiesto que de 10 diferentes inmunoanálisis de testosterona evaluados, ninguno presentaba un comportamiento analítico adecuado en el rango de valores esperables en niños y mujeres, cuestionando gravemente su utilidad clínica en muchos escenarios [6, 7]. Estos hallazgos en los análisis de testosterona fueron replicados en otros estudios [8] y extendidos a otros analitos como el estradiol. Los estudios de Lee et al. y Stanczyk et al. [9, 10], tomando también como referencia la espectrometría de masas, muestran la incapacidad de los inmunoanálisis para cuantificar de forma precisa y exacta las concentraciones de estradiol en muestras de suero de mujeres post-menopáusicas.

Ante esta dificultad para medir la concentración de esteroides, entre ellos la testosterona, con la exactitud, reproducibilidad y sensibilidad necesarias, la *Endocrine Society* de los EE. UU., se posicionó en 2007 [11] recomendando el uso de métodos basados en Espectrometría de Masas como método de referencia para la medida de la concentración circulante de testosterona. Asimismo, en 2010, la *Endocrine Society* de los EE. UU. junto con los *Centers for Disease Control and Prevention* (CDC) realizaron una declaración de consenso con el objetivo de estandarizar los procedimientos de medida de la testosterona [12]. A partir de estos posicionamientos, los CDC iniciaron un proyecto para la estandarización de la medida de la concentración de testosterona denominado *Hormone Standardization* (HoSt) Program [13], con el objetivo de mejorar la reproducibilidad, el sesgo y el error total de la medida de la concentración de testosterona y su comparabilidad. Finalmente, en 2013 [14, 15] se publica el desarrollo y validación de un procedimiento de LC-MS/MS calibrado con el material SRM 971, material de referencia trazable y conmutable preparado por la NIST (*National Institute of Standards and Technology*) para la medida de la testosterona. Los resultados de seguimiento en los últimos años del programa de estandarización de los CDC muestran de forma consistente que, aunque se observan mejoras en algunos métodos de inmunoanálisis, las técnicas basadas en espectrometría de masas son las que presentan mejores resultados.

De forma análoga, la evidencia acumulada de las limitaciones de los inmunoanálisis para estradiol ha conducido a la creación de sinergias entre laboratorios, sociedades científicas y fabricantes con el objetivo de intentar mejorar los métodos analíticos existentes y facilitar su estandarización. El mismo programa HoSt del CDC cubre el análisis de estradiol en suero evaluando periódicamente el comportamiento de los distintos ensayos [16]. Los análisis muestran de forma consistente que las técnicas basadas en espectrometría de masas son las que presentan mejores resultados, sin observarse, sin embargo, mejoras significativas en el comportamiento analítico de los inmunoanálisis para estradiol [17]. El programa HoSt se ha expandido, además, a las mediciones de 25-hidroxivitamina D, al sufrir los inmunoanálisis los mismos problemas que con la medición de testosterona y estradiol.

## Consideraciones preanalíticas

Las condiciones preanalíticas son de extraordinaria importancia, independientemente del método de medida. Por ello, se han de seguir de forma escrupulosa.

La secreción de testosterona se ajusta a un ciclo circadiano, con valores máximos a las 8 h y mínimos a las 20 h. Las variaciones pueden llegar a tener una amplitud del 36% [18] por lo que la obtención de la muestra sanguínea para el análisis de esta hormona debe efectuarse por la mañana. Asimismo, la testosterona circula mayoritariamente unida a SHBG (unión de alta afinidad y baja capacidad) y albúmina (unión de baja afinidad y alta capacidad) y solo una pequeña fracción circula de forma libre llegando a los tejidos diana. Esta última fracción se transforma en las células diana en dihidrotestosterona, que tras unirse a su receptor citosólico se traslada al núcleo celular uniéndose a elementos de respuesta a andrógenos específicos en el ADN. Por ello, la fracción libre se relaciona más directamente con la acción de la dihidrotestosterona y tiene mayor relevancia para la clínica.

No obstante, la correcta medición de la concentración de testosterona libre constituye un reto para los laboratorios clínicos. En efecto, aunque existen inmunoanálisis comerciales, estos en general presentan importantes limitaciones por lo que se desaconseja su uso con fines asistenciales [19], [20], [21]. La metodología de referencia para la medición de testosterona libre son los métodos de ultrafiltración y diálisis de equilibrio que separan, previo al análisis mediante RIA o espectrometría de masas, la testosterona libre de la unida a proteínas. Sin embargo, estos métodos son muy laboriosos técnicamente y no están disponibles en la mayoría de los laboratorios clínicos. Alternativamente, la recomendación para la valoración de testosterona libre es su cálculo mediante ecuaciones que tienen en cuenta las concentraciones de testosterona total, SHBG y albúmina [22].

El estradiol circula unido a SHBG en un 95%. La concentración sanguínea de estradiol no exhibe un ritmo circadiano, pero cambia durante las distintas fases del ciclo menstrual, al igual que lo hace la testosterona. Por este motivo, la obtención de la muestra para la medición de ambas hormonas debe realizarse en la fase folicular temprana del ciclo menstrual en mujeres en edad fértil.

## Retos en el análisis. Ventajas y limitaciones de los inmunoanálisis

Una vez asegurada la fase preanalítica correcta, el valor clínico de los resultados de laboratorio depende en gran medida del uso de una metodología apropiada. Las mediciones en suero de esteroides sexuales con fines asistenciales y/o de investigación presentan retos analíticos importantes. Entre ellos destaca la gran diversidad existente de metabolitos estructuralmente parecidos (moléculas derivadas del colesterol) de forma endógena o también que pueden ser administrados de forma exógena, la existencia de un amplio rango de concentraciones de interés clínico y la presencia de formas circulantes libres y unidas a proteínas de transporte. Actualmente, los métodos de inmunoanálisis son los métodos de medida más ampliamente utilizados a pesar de estar limitados en muchas ocasiones por su susceptibilidad a alguno de estos factores de confusión, hasta el extremo que un consorcio de numerosas sociedades científicas estadounidenses se han posicionado a favor de su sustitución por técnicas de análisis basados en espectrometría de masas, mucho más exactas [11, 12].

Los inmunoanálisis automatizados presentan las ventajas de ser prácticos y de permitir la medición de un elevado número de muestras de forma rápida y con bajo coste. Sin embargo presentan limitaciones como la falta de especificidad de los anticuerpos, que puede sobreestimar las concentraciones reales al medir moléculas parecidas al analito, una mayor posibilidad de que existan diferencias provocadas por la distinta matriz de las muestras de suero y de los calibradores, una mayor dificultad en la completa liberación de testosterona o estradiol de la SHBG y, en general, una sensibilidad limitada para medir de forma efectiva concentraciones bajas de esteroides.

La valoración de distintos inmunoanálisis para la medición de testosterona con el método LC-MS de los CDC mostró inexactitudes importantes para una concentración de 43.5 ng/dL (valores medios de sesgo por fabricante: Abott Architect 30%, Beckman Coulter 83–89%, Siemens −8.5–22.7%, Roche Cobas 48%, Tosoh Bioscience 37%). En esta misma concentración, situada en el rango medio-alto del intervalo de referencia para mujeres, los resultados reportados por los distintos inmunoanálisis estuvieron entre 34.4 y 87.8 ng/dL. La imprecisión de los inmunoanálisis mejoró para valores más altos (entre 160 y 534 ng/dL), con sesgos <20% en la mayoría de los casos [23]. En el caso del estradiol, el sesgo de 5 inmunoanálisis comerciales de uso habitual (Siemens Centaur; Siemens IMMULITE; Abbott ARCHITECT; Roche Cobas) en muestras de suero de hombres evidencia sesgos positivos del 11% al 74% en el percentil 25 (62 pg/mL) y del 12% al 53% en el valor medio (82 pg/mL) [24]. Uno de los constituyentes endógenos a los que se ha atribuido la posibilidad de interferir en el inmunoanálisis de testosterona y sobreestimar las concentraciones es el sulfato de dehidroepiandrosterona (SDHEA) [25]. Esta posible sobreestimación es especialmente crítica en las situaciones donde se pretende valorar concentraciones relativamente bajas, como ocurre por ejemplo en mujeres, niños y hombres en seguimiento de cáncer de próstata en los que la concentración de testosterona se utiliza como criterio de castración quirúrgica o química [25, 26] Existen también reacciones cruzadas con otros metabolitos endógenos como 11-cetotestoterona o 11-hidroxitestosterona. Son conocidas también las reacciones cruzadas de los anticuerpos de los ensayos de testosterona con esteroides sintéticos como nandrolona, danazol o noretisterona [27]. De especial importancia, en el caso del estradiol, es la presencia de reacción cruzada en los inmunoanálisis con los fármacos inhibidores de la aromatasa como exemestano [28] (que también causa falsas elevaciones de androstendiona por similitud molecular). Ello tiene una gran repercusión clínica ya que la monitorización de la eficacia de estos fármacos es, precisamente, el análisis de estradiol. También se ha descrito la existencia de reacciones cruzadas con antagonistas de los receptores de estrógenos como fulvestrant [29]. Más recientemente, se ha descrito que el tratamiento oral con estradiol puede resultar en concentraciones falsamente disminuidas de estradiol cuando se mide por inmunoanálisis [30]. La existencia y el grado de una interferencia concreta en el análisis de esteroides sexuales depende de los anticuerpos usados en el inmunoanálisis concreto, existiendo diferencias importantes entre fabricantes.

## Aportación de la espectrometría de masas

Idealmente, las mediciones analíticas de estos compuestos deberían basarse en métodos que no fueran susceptibles a interferencias por otros analitos (nuevos tratamientos con anticuerpos monoclonales, fármacos biológicos, etc.) de estructura similar o que interfieren en el procedimiento de medida, a la vez que, con suficiente sensibilidad para cuantificar concentraciones bajas, como las esperables en determinadas situaciones fisiológicas (como acontece en las muestras pediátricas y muestras de mujeres) o patológicas.

**Figura 1:**
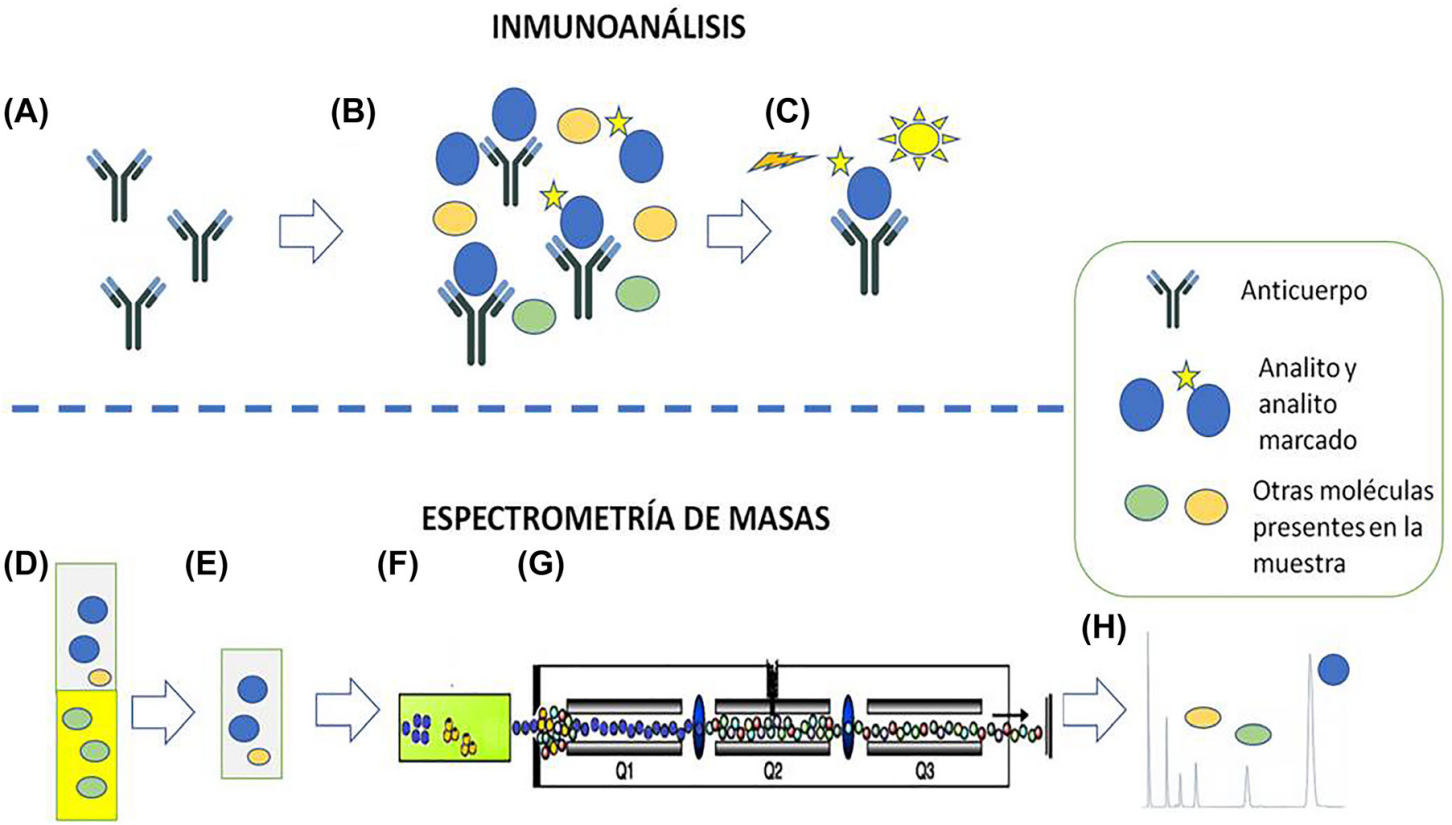
Inmunoanálisis. (A–C) Ejemplo de inmunoanálisis competitivo quimioluminiscente. (A) El método se basa en un anticuerpo específico que reconoce el analito de interés. (B) Se incuba el anticuerpo con la muestra (que contiene el analito de interés y otras moléculas) y con el analito marcado. Analito y analito marcado compiten por la unión al anticuerpo. (C) Se registra la señal quimioluminiscente procedente del analito marcado unido al anticuerpo. En el ejemplo, la señal registrada será inversamente proporcional a la cantidad de analito originalmente presente en la muestra. Espectrometría de masas (D–H). Ejemplo de cromatografía líquida y espectrometría de masas en tándem (LC-MS/MS). (D y E). Extracción de la muestra de suero con solvente orgánico. Este paso elimina posibles interferentes. (F) Separación de los componentes de la muestra mediante cromatografía líquida. (G) Selección de los iones específicos de los analitos. (H) Representación de los resultados. El área del pico cromatográfico es directamente proporcional a la cantidad de analito originalmente presente en la muestra.

En este sentido, la aparición de la espectrometría de masas posibilita superar las limitaciones de los inmunoanálisis en una gran mayoría de escenarios clínicos. Los fundamentos de ambas metodologías (inmunonálisis y espectrometría de masas) son distintos. En efecto, aunque existe una gran diversidad de técnicas de laboratorio basadas en inmunoanálisis y de espectrometría de masas, estas pueden resumirse en que los inmunoanálisis se fundamentan en reacciones antígeno-anticuerpo mientras que la espectrometría de masas no emplea reacciones antígeno-anticuerpo y se relaciona directamente con las características estructurales (masa y espectros de masa) de los analitos (Figura 1).

La importancia de la necesidad de usar metodología con comportamiento analítico adecuado para las mediciones hormonales se refleja también en el creciente consenso en las revistas científicas de mayor impacto en sus requerimientos para aceptar publicaciones. La revista *The Journal of Clinical Endocrinology and Metabolism* publicó un editorial en el que exigía, a partir del año 2015, el uso de espectrometría de masas en aquellos trabajos en los que los resultados de esteroides sexuales fueran un “*endpoint*” relevante [31]. Poco después, la misma revista publicó una *Letter of concern* valorando la complejidad del tema [32] y concretó unas instrucciones para los autores relacionadas con los requisitos de las determinaciones de hormonas esteroides [33].

**Figura 2:**
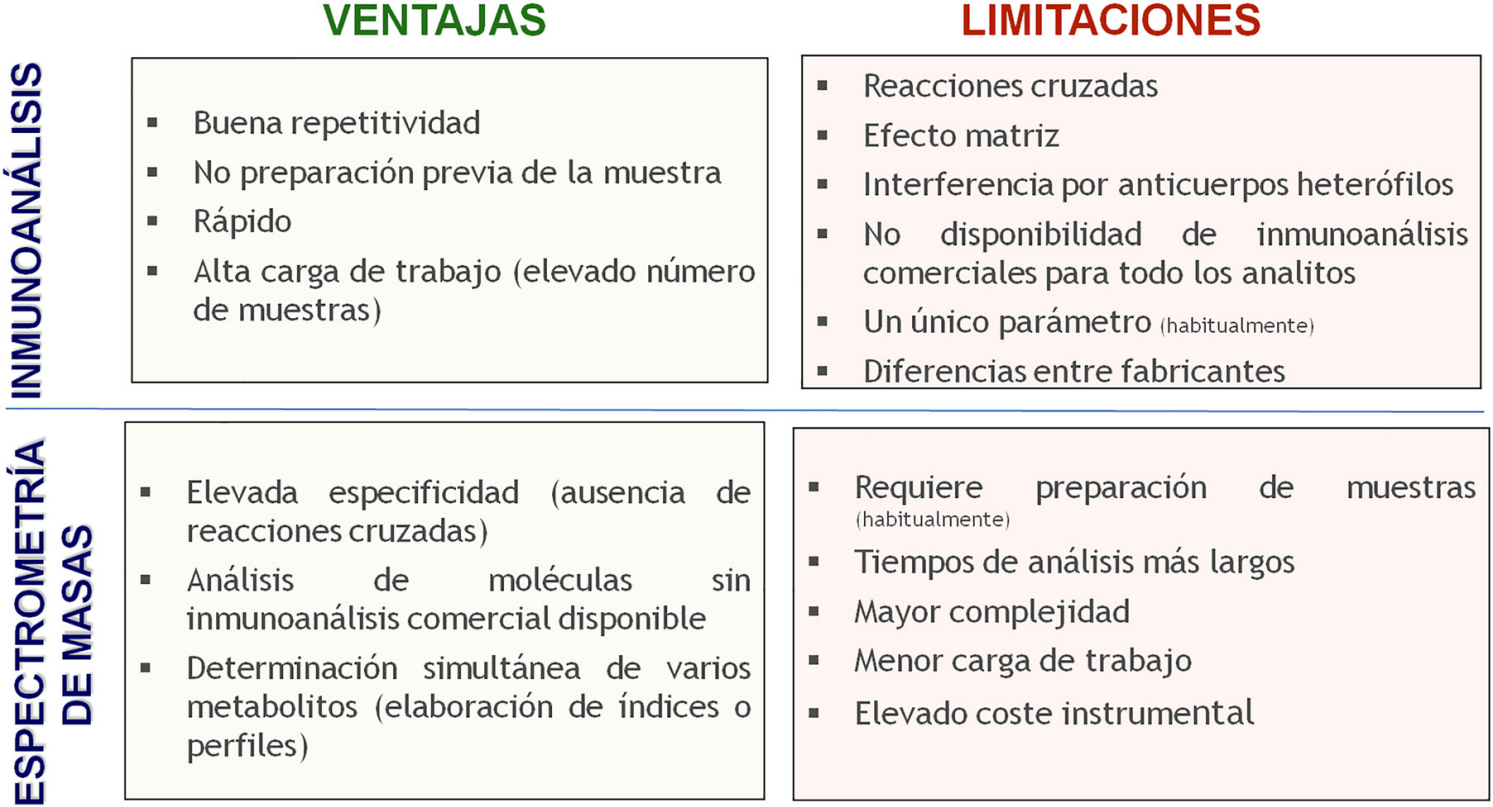
Ventajas y limitaciones de los métodos basados en inmunoanálisis y espectrometría de masas.

**Figura 3:**
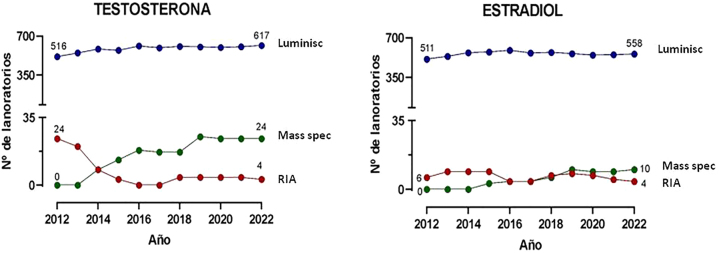
Número de laboratorios que proporcionan los resultados de testosterona y estradiol con inmunoanálisis quimioluminiscentes (Luminisc), espectrometría de masas (Mass Spec) y radioinmunoanálisis (RIA) en el programa de control de calidad externo europeo organizado por el Referenzinstitut für Bioanalytk (Alemania).

La Figura 2 compara las ventajas y desventajas de los inmunoanálisis quimioluminiscentes de uso habitual con los métodos basados en espectrometría de masas. Si bien los inmunoanálisis presentan una buena repetitividad y rapidez, que permite manejar de forma adecuada altas cargas de trabajo, la espectrometría de masas presenta una gran especificidad analítica (ausencia reacciones cruzadas por metabolitos endógenos o exógenos, ej. fármacos [27], [28], [29]), menor efecto matriz y ausencia de interferencia por anticuerpos heterófilos. Otras ventajas de la espectrometría de masas incluyen su versatilidad (posibilita por ejemplo medir casi cualquier metabolito) o la facilidad para la medición simultánea de varios metabolitos (posibilita elaborar perfiles o paneles a partir de una misma muestra). Además, dada su elevada especificidad, se minimiza la variabilidad inter-laboratorio, facilitando la elaboración de intervalos de referencia o puntos de corte de decisión médica comunes entre laboratorios. Sin embargo, la espectrometría de masas es una instrumentación relativamente costosa que requiere una considerable inversión económica, así como también requiere en general una preparación previa de la muestra y suele resultar en mayor complejidad (se necesita personal con una buena formación técnica) y mayor tiempo de respuesta. Ello probablemente limita su introducción masiva en los laboratorios clínicos. El análisis de los programas de control de calidad externo a nivel europeo permite observar su progresiva incorporación en los últimos años en algunos laboratorios de referencia clínicos a nivel europeo, la mayoría de los cuales mantenían los métodos de referencia usando RIA con extracción (Figura 3). En el programa UK National External Quality Assurance Service (UK NEQAS, año 2018), el 15% de los laboratorios participantes mide testosterona por espectrometría de masas [34]. En nuestro país la experiencia en incorporación de métodos de espectrometría de masas para mediciones hormonales distinta a los esteroides sexuales a nivel asistencial se centra especialmente en los últimos 3–4 años en los que diversos laboratorios han podido disponer de instrumentación dedicada a las hormonas.

## Situaciones clínicas en las que la espectrometría de masas presenta mayor interés

Tal y como hemos reseñado los ensayos de esteroides sexuales deben ser sensibles, específicos, exactos y precisos en un amplio rango de concentraciones [11, 12, 35, 36]. Hay algunas situaciones de especial relevancia en la práctica clínica que merecen una mención especial.

### Sensibilidad: medición de esteroides a bajas concentraciones

#### Medición de estradiol a bajas concentraciones

En el seguimiento de algunas situaciones especiales, por ejemplo, pacientes con cáncer de mama tratados con inhibidores de la aromatasa (en las que es necesario suprimir las concentraciones endógenas de estradiol), los ensayos deben poder distinguir entre concentraciones suprimidas de menos de 1 pg/mL [37] y concentraciones previas al tratamiento que en las mujeres menopáusicas suelen ser de 10 a 15 pg/mL. Del mismo modo, las mujeres con otras patologías como endometriosis o leiomiomas que se encuentran en esquemas de bloqueo y reemplazo (agonistas de GnRH para inducir la castración médica, junto con una dosis baja de estrógenos), no pueden ser controladas adecuadamente al no disponer de un sistema de medida específico, sensible y eficaz de medición de concentraciones bajas de estrógenos circulantes.

Además, los hombres y mujeres ancianos tienen concentraciones muy bajas de estradiol, en el rango de 5–30 pg/mL. En algunos casos, el control de estas concentraciones bajas podría ser útil; por ejemplo, en hombres con cáncer de próstata que se someten a una terapia de privación de andrógenos, las mediciones de estrógenos podrían ser útiles para evaluar esta terapia en objetivos como los huesos, el corazón y el estado metabólico [35].

En la misma línea, para evaluar el desarrollo puberal en niños, necesitamos medir con precisión los valores bajos de esteroides sexuales, particularmente en prepúberes y en pacientes con pubertad precoz, ya central, ya periférica, ya mixta, así como definir intervalos de concentraciones normales a lo largo de la infancia. En esta etapa de la vida, aunque es poco frecuente, algunos tumores gonadales secretan gonadotrofina coriónica humana, lo que lleva a una producción de hormonas sexuales que a menudo está por debajo del límite de detección del método [35, 38], [39], [40]. Además, la ginecomastia puberal en los niños, que implica un desequilibrio de testosterona y estrógenos, no se puede distinguir con los inmunoanálisis habituales [41].

#### Medición de testosterona

La mayoría de las pacientes que padecen síndrome de ovario poliquístico y otras formas de exceso androgénico no están siendo evaluadas adecuadamente debido a la falta de sensibilidad y/o especificidad de la mayoría de los inmunoánalisis actuales. Algunos estudios han demostrado que la testosterona libre se correlaciona mejor con la presentación clínica del síndrome de ovario poliquístico que la testosterona total [42, 43], aunque tal como se explicó anteriormente, la testosterona libre no está disponible en los laboratorios clínicos de rutina al ya que el método de referencia para su medición son los métodos de ultrafiltración o diálisis de equilibrio. Hay que destacar que las fórmulas que calculan la testosterona libre, requieren que la testosterona total haya sido medida con un método de precisión y, sólo en ese caso, ofrecen un rendimiento similar al del cálculo por ultrafiltración o diálisis de equilibrio. La Androgen Excess & PCOS Society recomienda desde 2009 el uso de la cromatografía líquida/espectrometría de masas tándem como método de referencia para la medición de testosterona total, y el cálculo de testosterona libre usando SHBG y albúmina, como método idóneo para estimar el hiperandrogenismo bioquímico en estas pacientes [44].

La evaluación de testosterona en mujeres con deseo sexual reducido podría ser informativa y es una necesidad clínica no satisfecha. Hay algunas pruebas, aunque controvertidas, en la que se sugiere una mejora el deseo sexual con el reemplazo de testosterona en mujeres con hipopituitarismo [45] o en mujeres premenopáusicas ovariectomizadas [46], pero los actuales métodos de medición no ofrecen suficiente confianza en los resultados de las concentraciones de testosterona tras la terapia sustitutiva por lo que no se recomienda.

Otras situaciones en las que se requieren métodos de alta sensibilidad son en el seguimiento de pacientes con cáncer de próstata sometidos a castración química para suprimir las concentraciones endógenas de testosterona, en la adolescencia para la evaluación de la pubertad temprana o tardía, así como tras el nacimiento durante la evaluación de la mini-pubertad en varones.

### Especificidad

Los pacientes pueden tener estrógenos/andrógenos circulantes derivados de fuentes exógenas, por ejemplo, hormonas esteroides sexuales de los alimentos, suplementos nutricionales, etc. [47]. Algunos de estos compuestos pueden reaccionar de forma cruzada con el anticuerpo en el inmunoanálisis y dar lugar a diagnósticos erróneos lo que conlleva un aumento considerable del gasto sanitario en pruebas innecesarias además de las molestias para el paciente.

### Exactitud

Finalmente, los resultados deberían ser comparables entre diferentes laboratorios. Los datos reproducibles son esenciales para el análisis y control del paciente cuyas pruebas son realizadas por varios laboratorios diferentes utilizando diferentes métodos. La introducción de técnicas correctamente estandarizadas en la mayoría de los centros hospitalarios evitaría que los pacientes seguidos en centros de referencia se vean obligados a trasladarse para una simple extracción de sangre, por falta de exactitud y reproducibilidad inter-ensayo entre centros.

## Recomendaciones del grupo de trabajo

En la medición de las concentraciones de esteroides sexuales, hay situaciones en las que la espectrometría de masas ha demostrado características metodológicas muy superiores a las del inmunoanálisis, especialmente cuando se requiere elevada sensibilidad y especificidad.

Mientras no exista la estandarización de todos los procedimientos de medida, se recomienda que los procedimientos utilizados en los laboratorios clínicos estén validados y cumplan las características metodológicas de reproducibilidad, sensibilidad y exactitud analíticas requeridas para medir las concentraciones, según las indicaciones de la guía CLSI [48] de la población a que se da servicio, y que cada laboratorio defina sus propios intervalos de referencia [49], [50], [51]. En este sentido, el programa HoSt del CDC ofrece sus servicios a laboratorios de todo el mundo con un coste perfectamente asumible. Por ello, consideramos muy recomendable recurrir a este programa desde los laboratorios del sistema nacional de salud para evaluar el rendimiento de los análisis empleados actualmente, y valorar su sustitución por otros ya estandarizados. Dicho programa indica el rango de medida que se certifica para cada método, proporcionando información valiosa de cara a valorar su utilidad en las distintas aplicaciones clínicas.

Aun cuando los inmunoanálisis pudieran mantener cierta utilidad para su uso en las mediciones de testosterona total en varones con concentraciones dentro del intervalo de referencia y de estradiol en mujeres premenopausicas, las recomendaciones de la última década indican la necesidad de usar métodos basados en espectrometría de masas para la medición de esteroides sexuales adecuadamente, y de forma muy particular en.

(1) Testosterona: las mediciones de la concentración de testosterona en el suero de pacientes pediátricos, mujeres y pacientes con neoplasias hormono-dependientes deben realizarse por métodos de elevada especificidad y sensibilidad analítica basados en espectrometría de masas. Si ello no es posible, deben considerarse aquellos inmunoanálisis que muestren un buen comportamiento analítico comparado con un método validado de espectrometría de masas como el ya valorado por el programa HoSt [52].
(2) Estradiol: las mediciones de las concentraciones de estradiol en el suero de pacientes pediátricos y en mujeres con cáncer de mama en tratamiento con inhibidores de la aromatasa debe realizarse con métodos de elevada sensibilidad y especificidad basados en espectrometría de masas en la valoración inicial y durante la monitorización del tratamiento. Los inmunoanálisis actuales no presentan sensibilidad adecuada para estas aplicaciones ni tienen la certificación del programa HoSt actualmente (Noviembre 2022) [52].

Entendiendo que no es viable actualmente usar las técnicas de medición basadas en espectrometría de masas en todos los centros hospitalarios nacionales, el grupo de trabajo recomienda introducir estas técnicas en al menos un centro por cada sistema de salud autonómico, para dar soporte adecuado a las situaciones clínicas descritas previamente y adquirir la experiencia necesaria para luego expandir paulatinamente estas técnicas al resto de la red hospitalaria, y adaptar de esta forma el sistema nacional de salud a las recomendaciones internacionales vigentes.
